# Statistical evaluation of surrogate endpoints: a systematic review

**DOI:** 10.1186/1745-6215-14-S1-P73

**Published:** 2013-11-29

**Authors:** Hannah Ensor, Robert Lee, Cathie Sudlow, Christopher J Weir

**Affiliations:** 1 Edinburgh MRC Hub for Trials Methodology Research, University of Edinburgh, Edinburgh, UK; 2 Division of Clinical Neurosciences, University of Edinburgh, Edinburgh, UK

## Background

Surrogate endpoints that can be used to predict treatment effect on an unobserved true endpoint are valued as their use may reduce the length, size or intrusiveness of a clinical trial. We aimed to update a previous systematic review on the theoretical statistical development of methods of validating surrogates for such a purpose.

## Methods

We searched MEDLINE and Web of Knowledge, from Jan 2003 to Feb 2013, locating 12,762 papers. After preliminary screening, two independent reviewers assessed each paper and 88 were agreed to contribute to the statistical methodology of surrogate evaluation.

## Results

Prentice's operational criteria, proportion of treatment effect explained and relative effect were the original techniques for evaluating surrogacy. These led to the development of several approaches for surrogate evaluation, namely; principal stratification, direct and indirect effects, meta-analytical (including the surrogate threshold effect) and information theory. Each methodological approach raises various computational issues in practice and conceptual issues regarding causality, transportability and interpretation.

## Conclusions

In theory, the principal stratification and direct and indirect effects approaches may be considered most appropriate because of their ability to validate surrogates on a causal basis. However, in practice, the well-established meta-analytical and information theory approaches are currently in advance of other techniques mainly due to superior ease of interpretation and computational feasibility.

